# A novel homozygous splicing variant in *FRA10AC1*: further delineation of the phenotype

**DOI:** 10.1038/s10038-025-01447-6

**Published:** 2026-01-23

**Authors:** Mohamed S. Abdel-Hamid, Ghada M. H. Abdel-Salam

**Affiliations:** 1 Medical Molecular Genetics Department, Human Genetics and Genome Research Institute, National Research Centre, Cairo, Egypt; 2 Clinical Genetics Department, Human Genetics and Genome Research Institute, National Research Centre, Cairo, Egypt

**Keywords:** Genetics research, Genetics of the nervous system

## Abstract

Biallelic variants in *FRA10AC1*, encoding a component of the spliceosomal C complex that is crucial for functional mRNA processing, have been recently associated with a neurodevelopmental disorder characterized by developmental delay, variable dysmorphic facies, growth retardation, and corpus callosum abnormalities. Skeletal and congenital heart defects were observed in some patients. To date, only 10 patients from 6 unrelated families with *FRA10AC1* variants have been reported in the literature. Herein, we describe a new patient harboring a loss-of-function variant in *FRA10AC1*. Our patient presented with global developmental delay, hypotonia, dysmorphic facies, hyperactivity, and severe intellectual disability. He had no seizures. Unusual findings noted in the patient were fused kidneys and bilateral cone dystrophy. Brain MRI showed a hypoplastic corpus callosum, delayed myelination, and a small cyst in the caudate nucleus. Exome sequencing revealed a novel homozygous variant in the donor splice site of exon 7 of the *FRA10AC1* (c.465+1G>A) as the likely cause of the patient’s phenotype. Thorough investigations of the exome data did not reveal any additional potential pathogenic variants to justify the patient’s renal and ocular phenotypes. The identified c.465+1G>A was confirmed by studying the patient’s mRNA to result in exon skipping and early protein truncation, p.(Trp127CysfsTer8). Our study increases the number of patients and variants associated with *FRA10AC1-*related disorder and contributes to increasing our understanding of *FRA10AC1* role in neurodevelopmental disorders. In addition, it reinforces kidney and ocular anomalies as part of this multisystem disorder.

## Introduction

Spliceosomopathies are genetic disorders resulting from defects in the spliceosome, an RNA-protein complex responsible for recognizing and removing noncoding introns from precursor mRNA (pre-mRNA). This process is essential for proper gene expression and regulation [1]. *FRA10AC1* (OMIM #608866) is located on chromosome 10q23.3 within the folate-sensitive fragile site FRA10A, comprises 19 exons and spans approximately 33 kb [2]. The encoded protein is a peripheral component of the spliceosomal C complex that interacts with DGCR14 (DiGeorge syndrome critical region 14) to ensure accurate splice-site recognition and correction of non-canonical or erroneous splice signals [3]. Although FRA10AC1 is not a core spliceosomal protein, it likely contributes to coupling transcription and splicing processes [4].

Biallelic variants in *FRA10AC1* were initially associated with neurodevelopmental delay, dysmorphic facies, growth retardation, and corpus callosum anomalies [5]. Later on, in-depth studies showed multisystem involvement [6, 7]. Functional studies revealed markedly reduced or absent *FRA10AC1* mRNA and protein in patient-derived fibroblasts, consistent with degradation through nonsense-mediated decay. In vitro assays further supported that FRA10AC1 loss primarily affects protein stability while maintaining nuclear localization and its interaction with DGCR14. Transcriptome-wide analyses indicated that global splicing integrity remained largely preserved, with only minor alternative splicing changes affecting a limited number of genes such as *SRGAPC2*, *TBC1D31* and *TMEM63A* [5].

Further evidence by Banka and co-authors [7] corroborated these observations, showing complete loss of *FRA10AC1* mRNA and protein expression in affected individuals, alongside restricted differential splicing and subtle transcriptional dysregulation involving developmental and chromatin-related genes. Together, these results indicate that *FRA10AC1* deficiency primarily leads to transcript and protein loss with minimal global disruption of pre-mRNA splicing, supporting nonsense-mediated decay as the main pathogenic mechanism.

Herein, we report an additional patient with *FRA10AC1*-related neurodevelopmental disorder and further delineate the clinical heterogeneity of this multisystem disorder. In addition, we compare our results with the existing literature and explore potential genotype-phenotype correlations.

## Materials and methods

### Patient

**Fig. 1:**
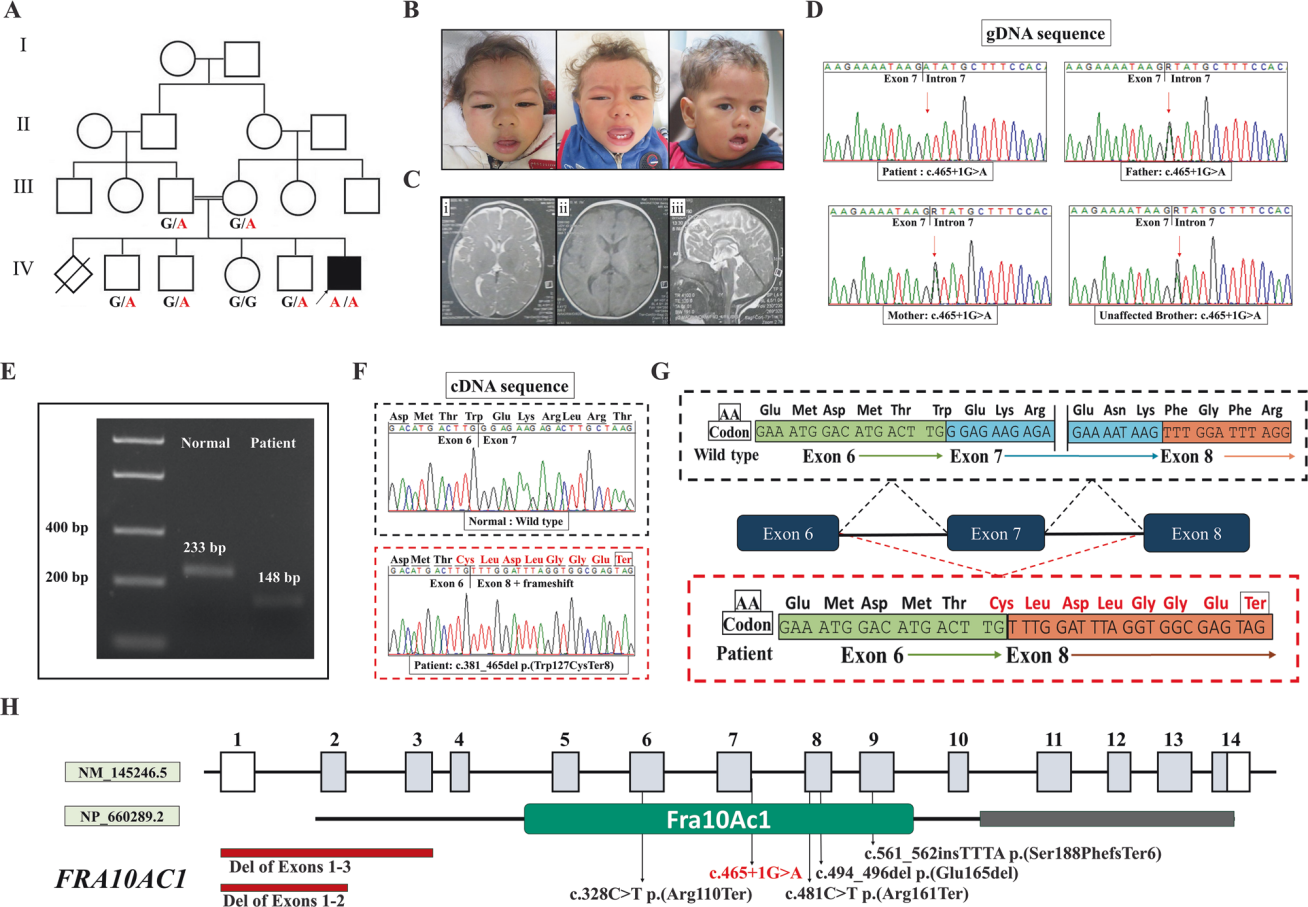
**A** Pedigree of the family. **B** Photographs of the face of our patient at the ages of 8 months, 1 year, and 4 years, showing a long face, bitemporal recession of hairline, high forehead epicanthic folds, long palpebral fissures, depressed nasal bridge, rounded bulbous nose and long philtrum. The face became rounded, and the long palpebral fissures became less prominent with age. **C** Brain MRI at the age of 8 months showing a small cyst in the caudate nucleus (i), delayed myelination (ii) and hypoplastic corpus callosum (iii). **D** Portions of the sequencing electropherograms (gDNA) showing the segregation of the *FRA10AC1* variant identified in the family. The arrow indicates the site of the variant. **E** A 2% agarose gel showing partial amplification of the cDNA of the *FRA10AC1* (from exons 6 to 8) in the patient and a healthy control individual. **F** Part of the sequencing electropherograms of the cDNA fragment of the patient in comparison to a healthy control individual. **G** Schematic diagram showing skipping of exon 7. **H** Schematic diagram showing the *FRA10AC1* exons and domains, and all reported variants, including our new one (in red)

Our patient was the sixth born of a consanguineous couple and a product of an uneventful pregnancy and full-term cesarean section (Fig. 1A). The first pregnancy ended with an unexplained intrauterine fetal death in the 24th week of gestation. There was no family history of note. His birth weight was 3000 g (Z score = –1SD), and he was admitted to the NICU because of respiratory distress. He was referred to our clinic at the age of 8 months because of global developmental delay. On physical examination at that age, weight was 6300 g (Z score = –2.2 SD), height was 70 cm (Z score = –1SD), and occipitofrontal circumference (OFC) was 43.5 cm (Z score = –2.3 SD). There was no visual contact or reaction to auditory stimuli. He did not smile or fixate, never babbled or vocalized. He was immobile and did not develop head support. His neurological examination revealed severe generalized hypotonia with truncal ataxia. His deep tendon reflexes were preserved. In addition, he had nystagmus. The boy had a long face, bitemporal recession of hairline, high forehead, epicanthic folds, long palpebral fissures, depressed nasal bridge, rounded bulbous nose and long philtrum (Fig. 1B). Epileptic seizures were not observed. Ophthalmologic assessment showed bilateral cone dystrophy, while the hearing test was normal. Abdomino-pelvic sonar showed fused kidneys at a low position at the midline of the pelvis. Brain magnetic resonance imaging (MRI) at the age of 8 months showed a small cyst in the caudate nucleus, periventricular white matter, delayed myelination and hypoplastic corpus callosum (Fig. 1C). We re-evaluated him at the age of 4 years. His weight, height and OFC were 13 kg (Z score = –1.9 SD), 95 cm (Z score = –1.4 SD) and 48.5 cm (Z score = –2.4 SD), respectively. At that age, he was able to stand and could say 3–4 monosyllable words. His developmental quotient was 35 (using Portage). Hyperactivity and agitation were noted. His face became rounded, and the long palpebral fissures became less prominent.

### Methods

The study was approved by the Medical Research Ethics Committee of the National Research Centre, Cairo, Egypt, in accordance with the Declaration of Helsinki, and written informed consent was obtained from the parents. Genomic DNA was extracted from the patient and all family members using the PAXgene Blood DNA Kit (Qiagen, Germany) according to the manufacturer's instructions. Exome sequencing was performed for the patient using the SureSelect Human All Exome 50 Mb Kit (Agilent, Santa Clara, CA, USA) and analyzed on Illumina NovaSeq 6000 (Illumina, San Diego, CA, USA). The obtained sequences were aligned to the UCSC human genome GRCh37/hg19, and variants were called using the GATK pipeline. Annotation of variants was performed using BaseSpace Variant Interpreter Server. Sanger sequencing was used to confirm the segregation of the variant in the parents and healthy siblings. To study the effect of the new *FRA10AC1* splice variant, total RNA was extracted from the leukocytes of the patient and a healthy control individual, followed by cDNA synthesis and sequencing (Supplementary Methods).

### Results

Exome sequencing identified a new homozygous splice variant in  the *FRA10AC1* (NM_145246.5: c.465+1G>A). This variant is located in the donor splice site of exon 7 of the gene. The variant was found in the heterozygous state in both parents, and the unaffected healthy siblings were either wild-type or heterozygous for the variant (Fig. 1D). The c.465+1G>A variant has a very low frequency in gnomAD v.4 (6 heterozygous individuals, MAF = 0.000003837). To investigate the effect of the variant on the splicing, we amplified exons 6 to 8 of the *FRA10AC1* using cDNA from the patient and a healthy control individual. Agarose gel electrophoresis revealed a shorter band in the patient compared to the control sample (Fig. 1E). Afterwards, sequence analysis confirmed that the variant induced skipping of exon 7 and early protein truncation p.(Trp127CysfsTer8) (Fig. 1F, G). According to ACMG classification guidlines of variants, the c.465+1G>A should be classified as a “pathogenic” variant (PVS1, PS3).

## Discussion

Biallelic variants in *FRA10AC1* have been identified as the cause of a neurodevelopmental disorder with growth retardation, dysmorphic features and corpus callosum abnormalities (MIM: # 620113). Ten patients from 6 unrelated families have been described to date with variable disease manifestations (Table 1) ranging from mild intellectual disability without motor delay to profound global delay associated with multiple congenital anomalies [5–7]. In this report, we describe a new patient and highlight the broad clinical manifestations and multisystem involvement associated with *FRA10AC1* variants.

**Table 1:** The clinical, brain imaging, and molecular characteristics of individuals with biallelic *FRA10AC1* variants

	von Elsner et al. (2022)					Banka et al. (2022)			Alsaleh et al 2022		This report
Individual	F1, P1	F2, P2	F3, P1	F3, P2	F3, P3	F1, P1	F1, P2	F2, P1	F1, P1	F1, P2	F1, P1
Sex	Female	Female	Male	Male	Male	Female	Female	Female	Female	Male	Male
Ethnicity	Arab (Egypt)	Arab (Saudi)	Arab (Egypt)	Arab (Egypt)	Arab (Egypt)	NA	NA	Arab (Saudi)	Arab (Saudi)	Arab (Saudi)	Arab (Egypt)
Significant prenatal and neonatal history	IUGR	Oligohydramnios, mild IUGR, hypotonia	Monozygotic twin died at 3 days	Respiratory distress	-	-	Feeding problems	Hypotonia, feeding problems	Respiratory distress	-	Respiratory distress, hypotonia
Birth weight (Z score)	−1.3	−2.7	−2.6	−4.2	−3.1	NA	NA	−5.1	−2	NA	–1
Age at last examination	3 y 1 m	9 y	15 y	10 y	7 y	23 y	21 y	12 y	6 y	1 y 3 m	4 y
Weight (Z score)	−3.4	−5.3	−3.9	−3.6	−1.8	Normal	–2	NA	−6.23	−2.36	–1.9
Height (Z score)	−2.8	−4.3	−4.8	−4.2	−2.9	5th centile	−3.6	NA	−6.47	+1.6	–1.4
OFC (Z score)	−4.1	−4.8	−3.8	−3.1	−2.0	3rd centile	–3.6	NA	–3.92	−0.08	–2.4
Craniofacial dysmorphism	Bitemporal narrowing, broad and medial flaring of eyebrows, upslanted and narrow palpebral fissures, hypertelorism, large mouth, retrognathia, full cheeks, low-set posteriorly rotated ears	High forehead, broad and medial flaring of eyebrows, narrow palpebral fissures, prominent nose, low-set ears	Long face, synophris, hypertelorism, narrow palpebral fissures, high arched palate, thick lips, pointed chin	Long face, synophris, hypertelorism, narrow palpebral fissures, high arched palate, thick lips, pointed chin	Long face, synophris, hypertelorism, narrow palpebral fissures, high arched palate, thick lips, pointed chin	Wide forehead, bushy eyebrows, blepharophimosis, epicanthus, long eyelashes, low-set ears, flat face, small nose, micrognathia, open mouth, short neck	Wide forehead, bushy eyebrows, blepharophimosis, epicanthus, long eyelashes, low-set ears, flat face, small nose, micrognathia, open mouth, short neck	Yes^a^	Protruding tongue, brachycephaly	Plagiocephaly, frontal bossing, high forehead, medial flaring of eyebrows, inverted epicanthus, strabismus, nystagmus, anteverted nares, pointed chin, prominent ears with simple antihelix	Long face, bitemporal recession of hairline, high forehead epicanthic folds, long palpebral fissures, depressed nasal bridge, rounded bulbous nose, long philtrum
Motor delay	Severe	Moderate	No	Mild	No	Mild	Moderate	Moderate-Severe	+ (Severe, nonambulatory)	Moderate	Severe
Cognitive delay	Profound	Profound	Mild	Borderline	Borderline	Moderate	Moderate	Moderate-Severe	Profound	Mild	Severe
Muscular hypotonia	+	+	+	+	+	+	+	+	+	+	+
Seizures	+	-	-	-	-	-	-	+	-	-	-
Behavioral problems	-	Autistic features and irritability	-	-	-	-	-	-	-	-	Agitation and nervousness
Speech	Non-verbal	Non-verbal	First words at 36 m	First words at 40 m	First words at 30 m	First words at 24 m	First words at 48 m	First words at 5 y	Non verbal	Non-verbal (babbles)	Non-verbal (babbles)
Regression	+	+	-	-	-	-	-	-	-	-	-
Brain anomalies	Agenesis of corpus callosum, mild hydrocephalus internus	Partial agenesis of corpus callosum, colpocephaly, unilateral retroorbital cyst	Thin stretched corpus callosum	Thin stretched corpus callosum	Thin stretched corpus callosum	NA	NA	NA	Thin corpus callosum, diffuse brain atrophic changes	NA	Hypoplastic corpus callosum, delayed myelination, caudate cyst
Skeletal anomalies	Clinodactyly of the 5th finger	Clinodactyly of the 5th finger	Clinodactyly of the 4th and 5th toes	Clinodactyly of the 4th and 5th toes	Clinodactyly of the 4th and 5th toes	Mild hypoplasia of metacarpal bones, short 5th finger	Clinodactyly of the 5th finger	Knee dislocations from the age of 9 y	-	-	-
Congenital heart disease	+	-	-	-	-	+		+	+	+	-
Kidney anomalies	+ (caudally located left kidney, increased cortical signal in both kidneys)	-	-	-	-	-	-	-	-	-	+ (fused kidneys at midline of pelvis)
Skin manifestations	Nevus flammeus on the forehead, nose and philtrum	Multiple brownish nevi are scattered on the nose and cheeks	-	-	-	-	-	-	-	Large hyperpigmented lesion in the lower back, atopic dermatitis	One café au lait patch on the arm, hypopigmented patch on the knee
Eye anomalies	-	-	-	-	-	Myopia, esotropia	Vision problems	-	Poor vision, strabismus	Nystagmus, strabismus	Nystagmus, cone dystrophy
Other anomalies	Echogenic foci in the liver	-	Growth hormone deficiency, anemia	Growth hormone deficiency	-	Hypothyroidism	Umbilical hernia, cleft palate, mild SNHL	Nissen fundoplication and percutaneous gastrostomy	Severe oropharyngeal dysphagia, tube feeding	Glanular hypospadias	-
*FRA10AC1* variants	Deletion of exons 1–2	c.561_562insTTTA p.(Ser188PhefsTer6)	c.494_496del p.(Glu165del)			c.328C>T p.(Arg110Ter)		Deletion of exons 1–3	c.481C>T p.(Arg161Ter)		c.465+1G>A p.(Trp127CysfsTer8)

*F* Family, *IUGR* Intrauterine growth restriction, *M* Month, *NA* Not available, *P* Patient, *SNHL* Sensorineural hearing loss, *Y* Year

^a^Not described in detail

Our patient had severe motor and cognitive delay, dysmorphic facies, hypotonia, and delayed speech, which were observed in all described patients. However, his growth showed improvement over time, indicating that growth retardation seems not to be a constant finding. In addition, the patient had behavioral problems, which were noted before in a single patient [5]. Although brain imaging findings were available for only 6/10 patients, corpus callosum abnormalities were strikingly observed in all of them. Similarly, our patient had hypogenesis of the corpus callosum, and we were able to show a small cyst in the caudate lobe of the basal ganglia.

Multiple congenital anomalies have been reported in patients harboring *FRA10AC1* variants [5–7]. Skeletal anomalies, congenital heart defects, and skin manifestations were found in 8/10 (80%), 5/10 (50%), and 3/10 (30%) of patients, respectively. Our patient did not have any skeletal or heart defects; however, he had a café au lait patch on the arm and a hypopigmented patch on the knee. In addition, the patient had fused kidneys at the midline of the pelvis. Similarly, one of the patients reported by von Elsner et al. [5] had a caudally located left kidney and increased cortical signal in both kidneys. Visual problems have been observed before in 4/10 patients [6, 7], however our patients showed cone dystrophy that appears to be the cause of the visual problems that have been reported before. Therefore, ophthalmological examination of patients with *FRA10AC1* variants is highly recommended. Other rare reported findings were seizures (2 patients), growth hormone deficiency (2 patients), sensorineural hearing loss (1 patient), cleft palate (1 patient), and hypospadias (1 patient).

The facial features (high forehead, long palpebral fissures, and bulbous nose) seen in our patient have been present in all individuals reported, suggesting that *FRA10AC1*-related neurodevelopmental disorder may be a recognizable syndromic disorder. Banka et al. [7] suggested that the facial dysmorphism was overlapping with Kabuki facial features but lacking the eversion of the lateral third of the lower eyelid. In our patient, the long palpebral fissures became less prominent with age. In addition, triangular  face and a pointed chin were noted to evolve with age [5, 6].

So far, six different variants have been described in patients with *FRA10AC1*-related disorder (Fig. 1H). These variants were 2 nonsense p.(Arg110Ter) and p.(Arg161Ter), 2 intragenic deletions (deletion of exons 1–2 and deletion of exons 1–3), 1 frameshift p.(Ser188PhefsTer6), and 1 in-frame deletion variant p.(Glu165del). None of these variants recurred in more than one family, though the majority of reported families were from Saudi Arabia (3 families) and Egypt (2 families). In this study, we identified the first splice variant (c.465+1G>A) in the gene, raising the total number of reported variants to 7. Functional characterization of this variant using the patient’s mRNA confirmed that it is associated with skipping of exon 7 and early protein truncation.

We are aware that it is early to comment on phenotype-genotype correlations based on the small number of patients reported to date (*n* = 11, including our patient). However, the three sibs harboring the in-frame deletion variant p.(Glu165del), which was confirmed to affect the FRA10AC1 stability but not its subcellular localization, had a milder phenotype in comparison to patients with loss-of-function variants [5–7]. None of the three sibs had skin, renal, ocular, or heart anomalies. In addition, they had mild intellectual disability with or without mild motor delay [5]. In contrast, patients with loss-of-function variants have more severe motor and cognitive delay and multiple congenital anomalies, as evident in our patient and those described before [5–7]. The identification of more patients with *FRA10AC1* variants in the future would greatly help to confirm this observation.

In conclusion, we reported a new patient with *FRA10AC1*-related disorder. Our patient shared the core clinical features of the disorder and had, in addition, kidney and ocular manifestations, which we suggest as an expansion of the multisystem involvement associated with the disorder. Phenotype-genotype correlations might exist as loss-of-function variants appear to be associated with a more severe cognitive and motor delay and multiple congenital anomalies. The diverse clinical phenotypes associated with pathogenic variants in *FRA10AC1* underscore the critical role of this gene in the neurodevelopmental pathways.

## Supplementary information

Supplementary Methods
