# Supplementary material for: A novel homozygous splicing variant in FRA10AC1: further delineation of the phenotype
Source: J Hum Genet. 2026 Jan 23;71(6):363–7. doi: 10.1038/s10038-025-01447-6 (PMC13199054; doi:10.1038/s10038-025-01447-6)
Supplement: Supplementary file 1 — Supplementary Methods [file 10038_2025_1447_MOESM1_ESM.docx]

**Supplementary Methods**

**Segregation of the *FRA10AC1*** **variant**

The *FRA10AC1* variant identified by exome sequence were confirmed in the parents and all family members using Sanger sequencing. The regions encompassing this variants (exon7) was amplified using the following primers sequence:

Exon 7F: 5’- ttccctccctgagtgaacca -3‘

Exo 7R: 5’- gacacatcataaacgcaggaca -3‘

Primers were designed by Primer3 software. PCR cycling conditions were: initial denaturation at 95°C for 5 min; 35 cycles of denaturation at 95°C for 30 sec; annealing at 62.5°C for 30 sec; extension at 72°C for 30 sec, and a final extension at 72°C for 5 min. PCR products were purified using ExoSAP-IT™ PCR Product Cleanup kit (Thermo Fisher Scientific, USA) and sequenced in both directions using the BigDye Terminator v3.1 Cycle Sequencing Kit ((Thermo Fisher Scientific, USA) and analyzed on the ABI Prism 3500 Genetic Analyzer (Applied Biosystems, Foster City, CA, USA) according to manufacturer's instructions.

**Functional study of the c.465+1G>A variant**

To study the effect of newly identified *FRA10AC1* variant (c.465+1G>A) on splicing, total RNA was extracted from the patient’s leukocytes and a healthy control individual using QIAamp RNA Blood Mini Kit (Qiagen, Germany). Five µg of total RNA were reverse transcribed into cDNA using QuantiTect Reverse Transcription Kit (Qiagen, Germany). The synthesized cDNA was then used as a template for partial amplification of the *FRA10AC1* gene (from exons 6 to 8) using one pair of primers: 5’- TTGGGGGAAAATGACAAGAC -3’ and 5’- CAGAAAAATTGACCTTTTCCTGA -3’ under the following conditions: 95°C for 5 min, a total of 35 cycles of 95°C for 30 sec, annealing at 62.5°C for 30 sec, 72°C for 30 sec, and a final extension of 72°C for 5 min. PCR products were separated by 2% agarose gel electrophoresis and then purified and sequenced as described above.
